# Triad-based screening risk assessment of the agricultural area exposed to the long-term PAHs contamination

**DOI:** 10.1007/s10653-018-0220-y

**Published:** 2018-11-22

**Authors:** Agnieszka Klimkowicz-Pawlas, Barbara Maliszewska-Kordybach, Bożena Smreczak

**Affiliations:** Department of Soil Science Erosion and Land Protection, Institute of Soil Science and Plant Cultivation - State Research Institute, Czartoryskich 8, 24-100 Puławy, Poland

**Keywords:** Ecological risk assessment, Lines of evidence, Triad approach, Polycyclic aromatic hydrocarbons, Contaminated area, Agricultural soil

## Abstract

The aim of the study was ecological risk assessment (ERA) of the agricultural soils located in the vicinity of the highly industrialized area and exposed to different emission sources of polycyclic aromatic hydrocarbons (PAHs). In this study, we demonstrated the combination of generic and site-specific ERA approach for screening assessment and delineation of the area of a high ecological risk. Generic approach was based on a hazard quotient and indicated that 62% of the research area needs further assessment. For site-specific evaluation, the Triad approach was utilized. Information from three lines of evidence (LoE): chemical, ecotoxicological and ecological, was integrated into one environmental risk (EnvRI) index. The chemical risk was derived from toxic pressure coefficients based on the total PAHs concentration. The ecotoxicological LoE included an acute toxicity testing: the luminescent bacteria *Aliivibrio fischeri* activity in both liquid- and solid-phase samples and the ability of crustacean *Thamnocephalus platyurus* to food uptake. The ecological LoE comprised microbial parameters related to soil respiration and enzymatic activity. Integrated EnvRI index ranged from 0.44 to 0.94 and was mainly influenced by high values of chemical LoE risk, while the ecotoxicological and ecological LoE indicated no or low risk. Due to the relatively high uncertainty associated with the contradictory information given by LoEs, there is the need to confirm potential risk in a tier 2 analysis.

## Introduction

Many organic and inorganic pollutants may be released into the soil as a result of anthropogenic activity and affect soil ecological functions (Swartjes 2011; Cachada et al. 2016). One group of chemicals of significant environmental importance are polycyclic aromatic hydrocarbons (PAHs) formed and released in all processes of incomplete combustion of fuels, such as wood, coal or diesel (Maliszewska-Kordybach et al. 2008; Holoubek et al. 2009; Xiao et al. 2014). The majority of PAH sources are of anthropogenic origin, mainly due to industrial emissions, solid waste incineration and exhaust emission (Tobiszewski and Namieśnik 2012; Duan et al. 2015). As hydrophobic compounds, PAHs may remain in the environment for a long time and suspended on the dust particles undergo long-range transportation (Holoubek et al. 2009; Xiao et al. 2014; Duan et al. 2015). The rural regions are often located in the vicinity of highly urbanized/industrialized areas and may be exposed to emissions of various pollutants. Agricultural land is under the pressure of PAHs contamination mainly from dry and wet atmospheric deposition (Maliszewska-Kordybach et al. 2008; Holoubek et al. 2009). The increased level of these hydrocarbons may cause adverse changes in the agricultural ecosystems, create unfavourable environmental conditions for soil organisms and decrease soil biodiversity (Maliszewska-Kordybach et al. 2007; Liu et al. 2017; Umeh et al. 2017). Additionally, contamination of agricultural soils may create a risk of transfer PAH compounds into the food chain. For this reason, ensuring maintenance of high quality of production and the safety of farm produce is critical for human health (Cao et al. 2013; Duan et al. 2015). This is particularly important in countries like Poland, where agricultural land covers a great part (46%) of the total territory (Maliszewska-Kordybach et al. 2008; Central Statistical Office 2017). For assessing the risk from chemical contamination, the ecological risk assessment (ERA) procedures are recently applied.

ERA is a multi-step process aimed to collect and analyse environmental exposure and effect data to estimate the risk of contamination to ecosystems (US EPA 1998; Sutter et al. 2000; Swartjes 2011; Karczewska and Kabała 2017). The first methodological guide of ERA was developed by the US EPA and over time has become a reference in the risk assessment process and was modified or adapted for managing polluted sites in many countries (US EPA 1998; Weeks et al. 2004; Ashton et al. 2008; Merrington et al. 2008; Perrodin et al. 2011; Swartjes et al. 2012; Terekhova et al. 2015). Two different types of ERA are reported in the literature: a predictive approach associated with the authorization of chemicals and derivation of safe levels of new substances before they are placed on the market, and a diagnostic approach enabling to assess adverse effects in already polluted areas and prioritization of contaminated sites before remediation (Solomon and Sibley 2002; Jensen and Mesman 2006; Perrodin et al. 2011; Cachada et al. 2016).

The risk assessment usually consists of three phases: a problem formulation (a site characterization with identification of main pollutants and their sources), an analysis phase (including exposure and effect assessment) and a tiered risk characterization (Sutter et al. 2000; Jensen and Mesman 2006; Gómez-Gutiérrez et al. 2007; Rutgers and Jensen 2011). Generally, the ERA methods include generic and site-specific assessments. For optimum protection of the soil ecosystem, ERA process should start with the application of generic and conservative assumptions (first tier risk assessment). This may be achieved by comparing predicted (PEC) or measured contaminant concentrations (MEC) in soil with national environmental quality standards (EQSs, e.g., soil screening levels or benchmark values) (Ashton et al. 2008; Dagnino et al. 2008; Rutgers and Jensen 2011; Swartjes 2011). The values of PEC (MEC)/EQS ratio > 1 indicate the existing ecological risk and are expressed as hazard or risk quotient (HQ or RQ) (Weeks et al. 2004; Hull and Swanson 2006; Merrington et al. 2008; Gutiérrez et al. 2009; Sorvari et al. 2013). For assessing the risk from the mixture of contaminants with the same mode of toxic action (like PAHs), it is possible to use the toxic equivalents (TEs) or toxic units (TUs) approach (Solomon and Sibley 2002; Xiao et al. 2014; Duan et al. 2015; Cachada et al. 2016).

Within the site-specific ERA, weight of evidence (WoE) methods (based on multiple lines of evidence—LoE) were proposed to characterize the risk and to determine possible ecological impacts (Hull and Swanson 2006; Semenzin et al. 2008; Cachada et al. 2016). The information obtained from different LoEs is used to conclude about an environmental system or stressors (e.g. pollutants). For terrestrial ecosystems, WoE approaches are still in an exploration and developing stage (Jensen and Mesman 2006; Rutgers and Jensen 2011). A specific type of WoE approach is the Triad method, a procedure originally developed by Long and Chapman (1985) to determine sediment quality. The Triad was recommended for the ERA of contaminated soils (Swartjes et al. 2012; Cachada et al. 2016) was included in the legislation in such countries like the Netherlands (Swartjes et al. 2012) and recently standardized by International Standardization Organization (ISO 19204 2017). The Triad approach includes data from three disciplines (LoEs): environmental chemistry (e.g. concentrations of toxic substances), ecotoxicology (e.g. bioassays) and ecology (ecological observations at the site) (Critto et al. 2007; Jensen and Mesman 2006; Semenzin et al. 2008; Rutgers and Jensen 2011). This multidisciplinary approach based on the combined evaluation of results from three LoEs allows the investigator to reduce the uncertainty associated with risk assessment (Critto et al. 2007; Rutgers and Jensen 2011). The Triad approach is usually performed as a tiered system. In each tier, the level of acceptable risk is assessed, which leads to a decision to conclude the assessment or proceed to the next ERA tier.

Recently, in the Environmental Protection Act in Poland new rules of contamination assessment have been introduced, based on the environmental risk assessment (Dz.U.^2016^.1395; Karczewska and Kabała 2017). EP Act of Poland was enacted with gaps in ERA due to limited available data and applications in contaminated land, and thus research in this field is still necessary. The main aim of the study was evaluation of ecological risk for agricultural soils located in the area exposed to different PAHs contamination/emission sources. The multidisciplinary Triad procedure was utilized for a site-specific risk assessment. We commenced from the evaluation of the contamination status of the research area and the first chemical screening based on the hazard quotient approach. The aim of this generic evaluation was to answer the question whether or not the ERA procedure is really needed. After deciding that ecological concern needs special consideration, the Triad simple screening including besides chemical data also ecotoxicological and ecological measurements was performed (Tier 1). The Triad method (screening phase) was used for the first time in Poland in evaluation of soils quality after flooding (Klimkowicz-Pawlas et al. 2012); however, to our knowledge, this study presents the first application of the Triad approach to assess the ecological risk for the agricultural soils exposed to the long-time contamination.

## Materials and methods

### Site characterization and soil sampling

**Fig. 1:**
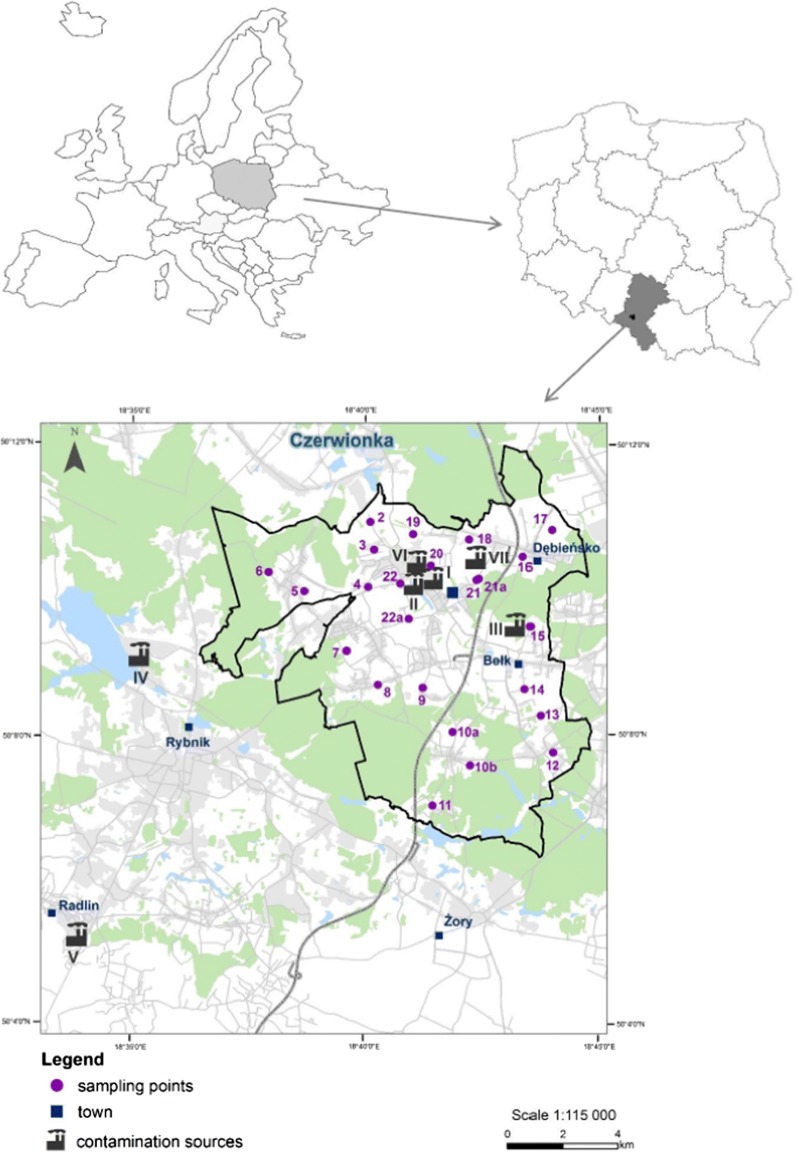
Location of sampling points and identification of potential pollutant sources (I—Debiensko coke plant, II—Debiensko hard coal mine and post-mining landfill, III—asphalt production plant, IV—Rybnik coke plant, V—Radlin coke plant, VI—Polho flotation waste recovery plant, VII—Zower coal recovery plant)

The study area is located in the Czerwionka municipality (Rybnik District) in the middle part of the Silesian Voivodeship in a distance of 40 km from Katowice and 45 km from the border with Czech Republic—Fig. 1. The territory covers an area of 114 km^2^, featured a high population density (357 persons/km^2^) and varied forms of land development, from densely urbanized land, industrial and post-industrial land to forested areas, fields and farms (Central Statistical Office 2015; Klimkowicz-Pawlas et al. 2017). The land usage structure is dominated by forested areas (42%) and arable land (34%); orchards, meadows and pastures cover around 10% of the total area. The crop structure is dominated by cereal crops with a small contribution root plants, oil plants and legumes (Chylak et al. 2008; Klimkowicz-Pawlas et al. 2017).

Geographically, the Czerwionka area is located in the south-western part of the Silesian Upland, within two smaller units of physical-geographical regions: Raciborska Basin, part of the Rybnik Plateau, and the Katowice Upland. The average height above sea level for studied area was 264 m (Table 1); the highest point is Ramża Hill (320 m a.s.l.), located in the north-west part of the study region. The climate in the region is influenced by colliding subtropical, the Arctic, and continental air masses. Average yearly precipitation and temperature are 719 mm and 8.9 °C, respectively. The annual distribution of winds is dominated by winds from the south-west, rather weak, with an average speed of 3.8 m s^−1^ (Table 1).

**Table 1:** Statistical evaluation of soil properties and meteorological data from Czerwionka area (*n *= 24)

Variable	Median	Mean	SD	Min	Max	LQ	UQ	CoV
Sand (%)	72.4	72.1	9.8	55.1	92.5	67.0	76.8	13
Silt (%)	25.5	25.7	8.9	7.3	40.8	21.6	30.4	35
Clay (%)	2.1	2.0	0.9	0.2	4.2	1.6	2.7	44
C_org_ (g kg^−1^)	14.4	11.9	8.9	5.2	40.9	9.8	13.6	62
TC (g kg^−1^)	19.2	14.1	13.4	6.3	58.4	11.8	20.4	70
pH_KCl_	5.1	5.3	0.9	3.7	6.8	4.5	5.6	17
N_t_ (g kg^−1^)	0.6	0.6	0.3	0.3	1.6	0.5	0.7	43
C:N	21.3	21.3	4.5	13.0	33.5	17.7	23.5	21
CEC (cmol + kg^−1^)	7.5	6.4	3.3	3.6	16.3	5.6	7.7	44
Temp (°C)	8.9	8.9	0.0	8.8	9.0	8.8	8.9	0.54
prec (mm)	716.7	719.3	10.9	705.2	740.8	710.0	729.5	1.52
etp (mm)	614.8	614.8	0.5	614.0	615.9	614.4	615.1	0.09
elev (m)	264.5	264.5	13.9	239.0	284.0	255.5	277.0	5.24
windsp (m s^−1^)	3.8	3.8	0.0	3.7	3.9	3.7	3.8	1.29

*Temp* average year temperature, *prec* average year precipitation; *etp* average potential year evapotranspiration, *elev* height above sea level, *windsp* average wind speed, *sand *content of fraction 2.0–0.05 mm, *silt* content of fraction 0.05–0.002 mm, *clay* content of fraction < 0.002 mm, *TC* total carbon content, *C*_*org*_ total organic carbon content, *N*_*t*_ total nitrogen content, *C:N* organic carbon-to-total nitrogen ratio, *CEC* cation exchange capacity, *SD* standard deviation, *Min* minimum value, *Max* maximum value, *LQ* lower quartile, *UQ* upper quartile, *CoV* variation coefficient (%)

Geologically, this area is located in the western part of Upper Silesian Coal Basin, region rich in natural resources, which include: a hard coal, a rock salt and a sand for construction (Chylak et al. 2008; Chylak and Kulikowski 2013). The soil cover in the study area is dominated by Cambisols (42%) and Luvisols (26%) with small contributions of Phaeozems, Fluvisols and Histosols (7–11%). Western and southern parts of the Czerwionka region are a part of the Landscape Park “Cistercian Landscape Compositions of Rudy the Great”, which combines the Vistula and the Odra rivers and was created in order to protect the ecological corridor of European importance, enabling migration of many animals species (Chylak and Kulikowski 2013).

Twenty-four soil samples were collected from the surface layer (0–30 cm) of agricultural land (mainly arable fields—75%) after the vegetation period and characterized in details (Fig. 1; Table 1). The sampling points were evenly spread and aimed to reflect direct exposure of soils to local and transboundary PAHs emission sources, different soil and hydrological conditions and diverse plant growing (Fig. 1). Each sample was a composite of about six subsamples collected from an area of 1 m^2^. Soil materials were hand-mixed on site to homogenize, transported to a laboratory and air-dried at a temperature of 20 ± 2 °C, sieved to pass a 2-mm sieve-mesh and stored in the dark at a temperature of 16–18 °C before chemical analysis. Soil samples for biological activity measurements and for ecotoxicological tests were prepared according to ISO 10381-6 (1993) method.

### Potential contamination sources description

The north part of Czerwionka region is dominated by industrial areas: a mine water desalination plant, a cokery Debiensko, a Debiensko hard coal mine and an energy enterprise. In the east part of the region, an asphalt production plant and the A1 highway are located (Fig. 1). The main emission/contamination source in the area is a coke plant Debiensko, which has been operated over 100 years, since the beginning of the last century (1913). The coking plant achieved the full capacity at the end of the 1970s (up to 500 thousand tons of coke per year) and nowadays produces high-quality domestic coke sold in the country and in the foreign markets. The Debiensko hard coal mine was established at the end of the nineteenth century, and hard coal was extracted until 2000, when the mine was closed. The total area of mining was 45.5 km^2^. Mine wastes (34 million tons of solid wastes and 3 million tons of slurries) were deposited on the spoil heap and tailing ponds of the total area 140 ha (Grzesik and Mikołajczak 2008; Chylak and Kulikowski 2013). Wastes deposited on the mine tips are recently subjected to secondary exploitation, aiming the full recovery of coal contained in waste. The flotation waste recovery plant (Polho) exploits slurries deposited in ponds and produces floto-concentrates of coal. Coarse wastes deposited on the flat heap are exploited by the coal recovery plant—Zower (Chylak et al. 2008; Grzesik and Mikołajczak 2008).

The soil quality of the Czerwionka area is also influenced by the activity of neighbouring plants (e.g. coke plant Radlin and power plant located near Rybnik) and pollutant inflow from transboundary emission sources (e.g. the Ostrava-Karvina region in Czech Republic). The main part of industrial factories activity in the region is connected with energy production (coal and coke production), which may result in the emission of different pollutants, e.g. heavy metals, volatile products and PAHs, and influence the agricultural regions located in the vicinity of factories. Our previous studies (Maliszewska-Kordybach et al. 2010; Klimkowicz-Pawlas et al. 2017) reported the relatively high level of PAHs emission in this region, and thus these compounds were identified as the contaminants of potential concern associated with the site. Consideration of PAHs fate and transport suggested that they would be present in soils, with possible leaching to groundwater and create risk to soil habitat and retention functions.

### General physical and chemical characterization—chemical line of evidence (Chem-LoE)

The content of 16 PAH compounds from US EPA List was analysed based on the method previously described by Maliszewska-Kordybach et al. (2008) and Klimkowicz-Pawlas et al. (2012). Briefly, after addition of deuterated PAH compounds (d_8_-naphthalene, d_10_-acenaphthene, d_10_-phenanthrene, d_12_-chrysene, d_12_-perylene), soil samples were extracted with dichloromethane in ASE200 Accelerated Solvent Extractor (Dionex Co.). Water-free extracts were concentrated, cleaned up on glass mini-columns with activated silica gel and eluted with a mixture of dichloromethane/n-heksane. The concentration of PAHs was determined by gas chromatography with MS detection using an Agilent GC–MS apparatus (Agillent Technologies, Santa Clara, CA) equipped with DB-5 MS + DG fused-silica capillary column (J&W Scientific, USA). A certified reference material (CRM 131), laboratory control sample and solvent blank sample were applied for quality control. The recovery of individual PAHs from CRM 131 was within 62–84%, and precision expressed as a relative standard deviation (RSD) was below 12%. Method detection limit (MDL) for individual PAHs ranged from 0.53 to 2.37 μg kg^−1^, while method quantification limit (MQL) varied from 1.56 to 7.10 μg kg^−1^.

The soil particle size distribution was established by a laser diffraction method, using Mastersizer 2000 apparatus with Hydro MU attachment (Malvern Company) (Debaene et al. 2014). Total carbon content (TC) was measured after dry combustion (ISO 10694 1995) in Vario Macro Cube CN analyser (Elementar Analysensysteme GmbH). Total organic carbon (C_org_) content was determined by sulphochromic oxidation (ISO 14235 1998), the pH by the potentiometric method in 1 mol L^−1^ KCl solution (ISO 10390 2005), and total nitrogen content (N_t_) by Kjeldahl method (ISO 11261 1995). The cation exchange capacity (CEC) was calculated as the sum of extractable acidity (PN-R-04027 1997) and base saturation (K^+^, Na^+^, Ca^2+^ and Mg^2+^ cations).

### Ecotoxicology tools for screening assessment (Ecotox-LoE)

Three bioassays were utilized for the assessment of soil ecotoxicity. The Microtox^®^ 81.9% Screening test and Rapidtoxkit were applied for testing soil elutriates, and the SPT-Microtox for testing solid samples. The elutriates were prepared according to ISO 21268-1 (2007) method by mixing the soil with 0.001 mol L^−1^ solution of CaCl_2_ (soil/solution = 1:2), shaking for 24 h (125 rpm, 20 °C), centrifugation (15 min at 3000 rpm) and filtration with the 0.45 μm syringe filter. Ecotoxicity testing was performed in duplicate.

The Rapidtoxkit is a short ingestion test with anostracan crustacean *Thamnocephalus platyurus* as a test organism (Nałęcz-Jawecki et al. 2011). Organisms were hatched after 34-h incubation of dormant cyst at 25 °C under continuous illumination (3000–4000 lx). The larvae of *T. platyurus* were transferred into test tubes filled with soil elutriates (5 ml), and after 1-h incubation (25 °C, at darkness) the suspension of red microspheres was added as an artificial food. The crustacea fed on red latex beds for 30 min, and then they were fixed with Lugol solution. The number of intoxicated larvae was counted under the stereomicroscope. Stressed organisms do not take up the red particles or ingest them at a much lower rate, and thus the organism was counted as intoxicated, when its digestive tract was colourless, without any red particles. The effect of tested soil elutriates was expressed as inhibition of particle uptake.

Two different Microtox tests (Screening and Solid-Phase test—SPT), using bioluminescent marine bacteria *Aliivibrio fischeri* (formerly known as *Vibrio fischeri*) as test organisms, were performed. In the Microtox, 81.9% Screening test bacteria were exposed to the soil leachates for 15 min, while in the SPT-Microtox—to the soil suspension in the dilution series for 20 min. Changes of bioluminescence were measured on a Microtox Model 500 analyser (MicrobicsCorporation 1992). More detailed description of SPT-Microtox test was given in Klimkowicz-Pawlas et al. (2012).

### Ecology tools for screening assessment (Ecol-LoE)

Two soil microbial parameters: substrate induced respiration (SIR) and dehydrogenases activity (DHA) were determined for the characterization of the ecological line of evidence (Ecol-LoE) at screening level of ERA. DHA was measured by the method of Casida et al. (1964) using triphenyltetrazolium chloride (TTC) as an electron acceptor. After incubation of soil samples (24 h, 37 ± 2 °C), the formed triphenylformazan was extracted with ethanol, and the intensity of the red colour was measured at 485 nm wavelength using Lambda 45 UV–VIS spectrophotometer (Perkin Elmer, USA). SIR was determined according to the ISO 14240-1 method by the quantification of the carbon dioxide evolution after addition of readily degradable substrate. Field-moist soil samples (20 g) were placed in gas-tight jars, amended with a glucose (10 g kg^−1^), and incubated at 20 ± 2 °C for 6 h. The amount of glucose was established experimentally. The released CO_2_ was absorbed in 0.05 mol L^−1^ NaOH, and after back titration of non-consumed NaOH with HCl, the amount of CO_2_ production was calculated. SIR and DHA measurements were taken in triplicate.

### Risk calculation and data analysis

#### Statistics

Statistical analysis was carried out using the Statgraphics Centurion programme (version XV, Statpoint Technologies). Basic statistical parameters such as median, mean, standard deviation (SD), range (Min, Max), lower (LQ) and upper (UQ) quartile, standardized skewness and kurtosis, and coefficient of variation (CoV) were determined. In cases where the PAHs content was below MQL, the MDL values were used for calculations. For the evaluation differences among contaminated soils and the reference soil, one-way analysis of variance (ANOVA) was applied, followed by the Duncan multiple-range test (significance at *p* ≤ 0.05 level).

#### First chemical screening

In order to determine the potential ecological risk, the generic approach based on the hazard quotient (HQ) evaluations was used (Eq. 1). This method is widely described in the literature (Weeks et al. 2004; Hull and Swanson 2006; Gutiérrez et al. 2009; Sorvari et al. 2013) and allows to indicate the magnitude of hazard that pollutants might pose to the soil organisms. The total concentration of each PAH measured in soil samples (*C*_*i*_ in µg kg^−1^) was compared with corresponding quality values (MPC_i_ in µg kg^−1^). In the present study, MPC_i_ was the corresponding maximum permissible concentration values of individual PAHs in soils according to Polish regulations (Dz.U.^2016^.1395). The hazard quotient was calculated as follows:

1 $${\text{HQ}} = \frac{{C_{i} }}{{{\text{MPC}}_{\text{i}} }}$$

To assess the potential additive effects of the PAHs mixture, the HQs for individual PAH compounds were summed to obtain the hazard index (HI)—Cachada et al. (2016).

#### Triad-based assessment (Tier 1)

The first screening stage of Triad procedure was applied in our study and included chemical (Chem-LoE), ecotoxicological (Ecotox-LoE) and ecological (Ecol-LoE) parameters. For quantitative evaluation of risk in tested soils, risk indexes were calculated according to the method described previously in details by Jensen and Mesman (2006), Sorvari et al. (2013) and Niemeyer et al. (2015). Results of all parameters measured for individual LoEs were used in the calculation. Different tests across the various LoEs were compared using a uniform scaling method. The magnitude of the risk were graded in an effect scale ranging from 0 (no risk) to 1 (highest risk). Risk indexes were calculated in relation to the reference soil, for which a risk value of zero was assumed. Calculation of integrated environmental risk (EnvRI) included the following steps: statistical analysis and scaling of results of each measured parameter within individual LoEs, combining the scaled information into the risk index for each LoE and integrating data from chemical, ecotoxicological and ecological LoE into one integrated environmental risk value (EnvRI).

For the Chem-LoE, the Toxic Pressure coefficient (TP) was calculated based on the total concentrations of 10 individual PAHs (Dagnino et al. 2008) and then the scaled information for each PAH was integrated according to a response addition model to give one chemical LoE risk value (ChemRI). For the Ecotox-LoE, the scaling of data was done based on the percentage of particle uptake (Rapidtoxkit) and bioluminescence inhibition (both Screening and SPT-Microtox tests) and obtained EcotoxRI risk index. For the Ecol-LoE, the risk (EcolRI) for the habitat function was calculated by scaling the data on soil microbial parameters (dehydrogenases activity and respiration). Finally, the indexes obtained from the 3 different Triad LoEs were combined in order to estimate EnvRI for each tested soil following the formula (Jensen and Mesman 2006; Niemeyer et al. 2015):

2 $${\text{EnvRI}} = 1 - \left( {10 ^{{[\log \left( {1 - {\text{ChemRI}}} \right) + \log \left( {1 - {\text{EcotoxRI}}} \right) + \log \left( {1 - {\text{EcolRI}}} \right)] / n}} } \right)$$

At the screening stage, equal weights were assigned to the individual LoE indexes, and therefore, the EnvRI (Eq. 2) was calculated as the mean value of three indexes. In order to assess the uncertainty in the estimation of EnvRI, Jensen and Mesman (2006) proposed evaluation of the standard deviation (SD) among the indexes for each LoE (ChemRI, EcotoxRI, EcolRI). The obtained EnvRI values were compared with specific thresholds depending on the calculated deviation (Jensen and Mesman 2006; Sorvari et al. 2013).

## Results and discussion

### Soil properties and selection of reference soil

The statistical evaluation of the soil properties is presented in Table 1. The basic physicochemical properties of soils were relatively uniform, with CoV ranging from 13 to 62% (Table 1). The soils from the study area were dominated by loamy sands (72.4% of sand, 25.5% silt and 2.1% of clay), were acidic (median pH_KCl_ 5.1), and reflected general characteristic of the majority of Polish agricultural soils (Maliszewska-Kordybach et al. 2008). They were not rich in organic carbon (median C_org_, 14.4 g kg^−1^; interquartile range 9.8–13.6 g kg^−1^) and nitrogen (0.3–1.6 g kg^−1^); C_org_ content corresponded to the low European values according to the European Union criteria (Rusco et al. 2001). The C/N ratios were within the range of 13.0–33.5, with the average value of 21.3, which may indicate possible soil degradation (Obrist et al. 2015). Soil organic matter and clay content are the main properties controlling the fate, transport and retention of pollutants in soils (Semple et al. 2013; Cachada et al. 2016; Okere et al. 2017; Umeh et al. 2017). The low content of clay and organic matter (Table 1) promotes bioavailability/bioaccessibility of PAHs due to limited sorption sites, thus increasing the leaching and distribution of the contaminants to underground and surface waters (Duan et al. 2015; Riding et al. 2013; Semple et al. 2013; Ogbonnaya et al. 2014; Umeh et al. 2017). As a consequence, pollutants may impair soil functions and pose risk to soil organisms. In addition, soil acidity can regulate the sorption capacity of organic matter (Bucheli et al. 2004; Semple et al. 2013; Obalum et al. 2017), create stress conditions for soil microorganisms and thereby increase their sensitivity to pollution (Maliszewska-Kordybach et al. 2007; Suszek-Łopatka et al. 2016).

The selection of a proper reference soil is a key factor in risk assessment of contaminated sites and has to follow few general rules (Dagnino et al. 2008; Sorvari et al. 2013). An appropriate control soil would be a non-contaminated soil of similar physicochemical (e.g. organic matter content, pH, texture) and biological properties as tested soils (Dagnino et al. 2008; Gutiérrez et al. 2015). Based on the statistical evaluation of soils physicochemical properties (Table 1), the reference soil in our study was selected from a point where no elevated PAH concentrations were determined by chemical analysis. The PAHs concentration in reference soil were 408 μg kg^−1^ and corresponded to the values reported in typical rural areas of Poland and other European countries (Maliszewska-Kordybach et al. 2008; Holoubek et al. 2009; Klimkowicz-Pawlas et al. 2017; Okere et al. 2017). It was a loamy sand soil characterized by a low content of organic carbon (12.9 g kg^−1^) and acidic reaction (pH = 5.4). Physicochemical and biological properties of the reference soil reflected the average soil properties in the area (Table 1) and fulfilled the criteria described for the reference soil used in the ecotoxicological characterization of soils (ISO 15799 2003; Dagnino et al. 2008; Gutiérrez et al. 2015).

### Contamination status of the area and first chemical screening

The total concentration of Σ16PAHs ranged from the 376 to 5695 µg kg^−1^ dry weight with median value of 1271 µg kg^−1^ (Table 2) and was threefold higher than the median concentration of Σ16PAHs (395 µg kg^−1^) reported by Maliszewska-Kordybach et al. (2008, 2010) for the arable Polish soils. Average PAH content was comparable to values reported by Duan et al. (2015)—822 µg kg^−1^, Huang et al. (2016)—1280 µg kg^−1^ and Liu et al. (2017)—917 µg kg^−1^ for agricultural soils subjected to the long-time PAHs emission. The higher PAH concentrations (> 1000 µg kg^−1^) were found in samples collected from the central and north part of the Czerwionka region. The high content of hydrocarbons at these sampling sites might be attributed to diffuse and stationary PAH sources: high traffic volume, houses heating and intensive anthropogenic activity mainly coke production, coal mining, and nowadays mine wastes recovery (Grzesik and Mikołajczak 2008). High share (80%) of the high molecular weight PAHs (four to six rings) in total PAHs concentration implies a domination of combustion over petrogenic sources (Tobiszewski and Namiesnik 2012; Klimkowicz-Pawlas et al. 2017). More detailed analysis of PAH sources and their composition profiles can be found in Klimkowicz-Pawlas et al. (2017). Additionally, the region was characterized by relatively high values of the anthropogenic indexes as total dust emission and dust emission from industrial sources, which were 6827 and 1084 kg year^−1^ km^−2^, respectively (Central Statistical Office 2015; Klimkowicz-Pawlas et al. 2017). The lowest PAHs concentrations were observed in the south of the study region (part of the Landscape Park), where less traffic volume and no industrial activities were observed. Among 16 PAHs listed in US EPA, seven have been classified as probable human carcinogens (Σ7PAH); they are BaA, CH, BbF, BkF, BaP, IndPyr and DahA (US EPA 1993). Table 2 showed that the total concentration of Σ7PAH ranged from 181 to 2427 µg kg^−1^ with median value of 552 µg kg^−1^ and accounted for 43% of Σ16PAHs. Among the carcinogenic PAHs, CH, BbF and BaP were the most abundant compounds in soil samples and contributed to 15–21% in total 7PAHs. General characterization of the study area has shown relatively high level of PAH compounds in soil, which may affect the soil functions (e.g. retention and habitat) and reveal high risk for humans.

**Table 2:** The concentration (µg kg^−1^) of individual PAHs (*n* = 24)

PAH	Mean	Median	SD	Min	Max	LQ	UQ	CoV
Naph^a^	61	55	54	13	292	32	69	89
Acyn	9	5	9	<MQL	42	4	10	104
Acen	17	14	10	5	49	10	20	62
Flu	18	14	17	4	85	9	19	94
Phen	233	147	228	41	984	96	271	98
Anth^a^	31	17	30	<MQL	111	10	49	97
Fln	338	227	309	52	1156	132	431	91
PYR	251	163	234	34	884	93	329	93
BaA^a^	109	77	96	16	369	42	135	88
CH^a^	153	117	125	35	513	63	180	82
BbF^a^	145	109	110	44	443	64	169	76
BkF^a^	91	65	78	21	289	41	107	85
BaP^a^	126	85	116	22	441	51	150	92
IndPyr^a^	97	68	84	27	320	39	112	86
DahA^a^	19	17	13	6	59	10	22	69
BPer^a^	90	62	82	5	309	32	108	91
Σ16PAH	1787	1271	1515	376	5695	753	2143	85
Σ7PAH	741	552	618	181	2427	318	870	83
HMW	1419	1005	1237	293	4648	586	1706	87

*SD* standard deviation, *Min* minimum value, *Max* maximum value, *LQ* lower quartile, *UQ* upper quartile, *CoV* variation coefficient (%), < MQL values below the method quantification limit

^a^PAH compounds included in Polish regulations (Dz.U.2016.1395), 16 PAH content of 16 PAHs according to US EPA list (1995), 7PAH—content of 7 carcinogenic PAHs (BaA + Chr + BbF + BkF + BaP + IndP + DahA) according to the US EPA (1993), HMW high molecular weight PAHs (from Pyr to BPer)

**Fig. 2:**
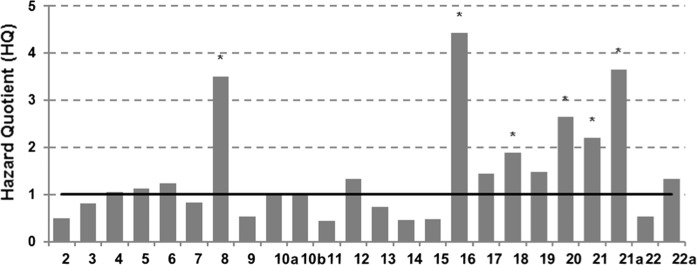
Delineation of the area of potential ecological risk; hazard quotient (HQ) values for the most abundant PAHs compound BbF; asterisks indicated sites HI values exceeded the Th value for 10 PAHs

To assess the potential ecological risk, the HQ approach was used in our study for the first chemical screening. The HQ values > 1 indicate existing ecological risk (Gómez-Gutiérrez et al. 2007; Gutiérrez et al. 2009; Sorvari et al. 2013; Cachada et al. 2016). In HQ calculations, 10 PAH compounds listed in Polish regulations (Dz.U.^2016^.1395) were taken into account, for which MPC values were 100 µg kg^−1^ (Naph, BaA, BaP, BbF, BkF, DahA) and 200 µg kg^−1^ (Anth, CH, BPer, IndPyr), respectively. Obtained HQs were in the range of 0.01–4.42, and most often the limit value was exceeded in the case of BbF (Fig. 2), one of the most abundant compounds (Table 2). It allows to conclude that over 60% of the study area might be under ecological risk (HQ > 1; Fig. 2) and needs to be subjected to a further detailed site-specific ERA evaluations and remediation (Dz.U.^2016^.1395). Therefore, HQ values determined for BbF were basis for making conclusions about possible risk at the research area. Since PAH compounds usually occur in soils as a mixture of different individuals, the hazard indexes were also calculated at each sampling site for the mixture of PAHs and were within the range of 1.92–22.72—data not shown. The threshold value (Th) for the HI for the ΣPAHs was assessed as 10 (Dagnino et al. 2008; Moreno-Jimenez et al. 2011), and obtained results generally were in line with data for individual hydrocarbons—Fig. 2. One of the principal goals of conducting the generic screening ERA was the identification of areas of special concern in the region, due to the occurrence of PAHs in soils. As was stated by many authors (Weeks et al. 2004; Hull and Swanson 2006; Gómez-Gutiérrez et al. 2007; Gutiérrez et al. 2009; Sorvari et al. 2013; Cachada et al. 2016), the HQ-based methodology may provide a good visualization of risk and is appropriate for the screening-level assessment. However, this conservative approach has many limitations. Assessment including only chemical evaluations may lead to an overestimation of risk since it has been recognized that total concentrations in soil are poor predictors of toxicity to soil organisms (Dagnino et al. 2008; Semenzin et al. 2008; Semple et al. 2013; Cachada et al. 2016; Umeh et al. 2017). Therefore, to more site-specific evaluations, ecotoxicological and ecological data were included in a further consideration.

### Ecological risk assessment—TRIAD-based screening assessment (tier 1)

Based on the first chemical screening, we found that soils from 15 sampling points (62.5% of the research area) need further assessment (Fig. 2). In order to better characterize the risk in the study area, the Triad screening assessment (Tier 1) was included in the evaluation. Triad method has been utilized until now to assess quality of bottom sediments (Gómez-Gutiérrez et al. 2007; Dagnino et al. 2008; Gutiérrez et al. 2009), groundwater (Crevecoeur et al. 2011), and in lesser extent for soil quality, mainly heavy metals contaminated soils (Karjalainen et al. 2009; Niemeyer et al. 2010, 2015; Ribé et al. 2012; Terekhova et al. 2015). There is no information on performing Triad-based ERA in soils contaminated with PAHs, especially in agricultural soils located in the vicinity of industrial sites. Till now, only data regarding application of ERA generic methods (HQ and TEF approach) are available for such areas (Cao et al. 2013; Duan et al. 2015; Huang et al. 2016; Liu et al. 2017).

In order to characterize the impact of pollutants on soil retention and habitat function, two types of bioassays were applied for an acute toxicity testing (Ecotox-LoE). Microtox test reflected the luminescent bacteria *A. fischeri* activity in both liquid- and solid-phase samples and Rapidtoxkit—the ability of crustacean *T. platyurus* to take up food (Nałęcz-Jawecki et al. 2011; Niemeyer et al. 2015). The response of the organisms depended on the tested species and in the case of *A. fischeri* the type of sample (elutriates or a bulk soil). Toxicity testing of soil elutriates revealed the high sensitivity of the *T. platyurus*—Table 3. Inhibition of the particle uptake in the Rapidtoxkit assay was in the range of 0.0 to 56.7% with an average of 34% (Table 3), which indicated slight acute toxicity of soil elutriates according to Foucault et al. (2013) criteria. Lower toxicity was observed in the Microtox Screening test, and bioluminescence was decreased in average of 16.5%—Table 3. Relatively low or zero toxicity in soil elutriates may indicate on the low mobility, extractability, and bioavailability of PAHs in tested samples as a result of ageing processes occurring in the long-time contaminated area (Bucheli et al. 2004; Holoubek et al. 2009; Niemeyer et al. 2010; Umeh et al. 2017). With ageing increases the desorption-resistant fraction of pollutants; desorption rates in historically contaminated soils are very slow such that the risk is acceptable (Riding et al. 2013; Semple et al. 2013; Umeh et al. 2017). Although organic matter may be low in most soils, carbonaceous materials as black carbon inherent in soils further retard bioavailability/bioaccessibility and desorption of PAHs upon ageing (Semple et al. 2013; Ogbonnaya et al. 2014). Our previous study (Klimkowicz-Pawlas et al. 2017) showed relatively high content (up to 45.3 g kg^−1^) of black carbon in soils from Czerwionka region. Regarding the SPT-Microtox, all soil samples were toxic and high inhibition of bioluminescence was observed (EC_50_ value was 4.8–36.4%, data not shown), which may indicate the high sensitivity of this test. However, as some authors (Klimkowicz-Pawlas et al. 2012; Foucault et al. 2013) pointed out, the EC_50_ from the SPT-Microtox test should be considered carefully in toxicity evaluation, since the bacteria response may be affected by soil properties (e.g. pH, C_org_ or clay content) which may result in the false increase in toxicity. In our study, the lowest EC_50_ values (high toxicity) noted in soils 6, 8, 18 and 22a were mainly related to the soil acidity (pH_KCl_ 3.9–4.9). Furthermore, the observed effect may result from the presence of other toxic agents in soils not analysed in this study.

**Table 3:** Statistical evaluation of ecological and ecotoxicological parameters (*n* = 24)

	Mean	Median	SD	Min	Max	LQ	UQ	CoV
Ecol-LoE								
DHA	33.9	32.8	15.6	13.1	86.9	22.4	38.2	46
SIR	9.0	8.0	5.8	2.6	26.3	5.1	10.2	65
Ecotox-LoE								
SPT-Microtox	91.2	91.0	6.9	77.6	99.8	85.7	98.0	7.5
Microtox screening	16.5	16.3	9.9	0.0	34.5	9.6	23.3	60
Rapidtoxkit	34.0	38.2	18.3	0.0	56.7	31.5	46.8	54

*DHA* dehydrogenases activity (µg TPF g^−1^d.m.), *SIR* substrate induced respiration (µg CO_2_ g^−1^d.m. h^−1^), SPT-and Microtox Screening (% of bioluminescence inhibition), Rapidtoxkit (% of particle uptake inhibition), *SD* standard deviation, *Min* minimum value, *Max* maximum value, *LQ* lower quartile, *UQ* upper quartile, *CoV* variation coefficient (%)

Ecological indexes (Ecol-LoE) comprised microbial parameters related to the soil respiration and enzymatic activity. Soil dehydrogenases activity and respiration intensity were relatively low with median value of 32.8 µg TPF g^−1^d.m. and 8.0 µg CO_2_ g^−1^d.m. h^−1^, respectively (Table 3). However, microbial parameters varied significantly within the tested soils (CoV 46–65%) and were related to soil properties, mainly the organic carbon content (correlation coefficient 0.44 and 0.76 at *p* ≤ 0.05 for SIR and DHA). Higher organic C levels in soil support higher microbial biomass and enzymatic activities, as Obalum et al. (2017) and Bielinska et al. (2018) noted that OC serves as carbon and energy source for most chemoorganotrophic microorganisms. Both tested microbial parameters reflect the metabolic activity of the whole microbial population (Muhlbachova et al. 2015; Bielińska et al. 2018) and are suggested as suitable indicators of soil quality, health and environmental risk (Jensen and Mesman 2006; Gutiérrez et al. 2015; Niemeyer et al. 2015; Bielińska et al. 2018).

The ecotoxicological LoE indicated the different risk levels when assays on soil elutriates were taken into account (Table 4). EcotoxRI indexes, based on the bioluminescence inhibition of *A. fischeri* in the Screening test, were in the range of 0.00–0.27 and revealed zero or low risk (only for 3 sampling points). Although *T. platyurus* was more sensitive species (Table 3), EcotoxRI index calculated for food uptake by the crustacean did not indicate a risk (Table 4). It probably resulted from the higher response of the *T. platyurus* in the reference soil in the Rapidtoxkit and confirmed previous findings of other authors (Sorvari et al. 2013; Gutiérrez et al. 2015) that selection of reference soil is a critical point in the ERA assessment. The risk values based on SPT-Microtox assay were slightly different and varied from medium to high risk (risk values from 0.51 to 0.96) for 4 sampling points (6, 18, 20 and 21 a)—Table 4.

**Table 4:** Risk values according to the chemical (Chem-LoE), ecotoxicological (Ecotox-LoE) and ecological (Ecol-LoE) lines of evidence

Sampling point	Chem-LoE	Ecotox-LoE	Ecol-LoE			
	TP	Microtox screening	Rapidtoxkit	SPT-Microtox	SIR	DHA
4	0.86	0.00	0.00	n.d.	0.10	0.38
5	0.86	0.00	0.00	0.00	0.54	0.23
6	0.90	0.20	0.00	0.96	0.31	0.10
8	1.00	0.24	0.00	0.00	0.33	0.12
10a	0.76	0.27	n.d.	n.d.	0.00	0.00
10b	0.84	0.07	n.d.	n.d.	0.12	0.49
12	0.89	0.19	n.d.	n.d.	0.61	0.39
16	1.00	0.07	0.00	0.00	0.00	0.00
17	0.93	0.01	0.00	0.00	0.00	0.01
18	0.98	0.07	0.00	0.82	0.71	0.10
19	0.95	0.27	0.00	0.00	0.00	0.00
20	1.00	0.07	0.00	0.51	0.07	0.09
21	0.99	0.18	0.00	0.00	0.00	0.00
21a	1.00	0.04	0.00	0.74	0.36	0.00
22a	0.88	0.00	0.00	0.00	0.00	0.44

For each sampling point values are scaled from 0 to 1 and are given in the relation to the reference soil (risk for reference soil is set to 0), *TP* toxic pressure coefficient, *SIR* substrate induced respiration, *DHA* dehydrogenases activity, *n.d.* not determined

The ecological LoE indicated no risk in 40% of investigated soils, and risk values calculated for DHA ranged from 0.01 (no risk) to 0.49 (low risk); meanwhile, the SIR-based risk was slightly higher (risk values 0.07–0.71) and indicated the medium risk only in soils from three sampling points (5, 12 and 18).

Chem-LoE was based on the toxic pressure calculation; obtained TP values were in the range of 0.76–1.00 and indicated on the high risk caused by PAHs. Extremely high risk (risk value = 1.00) was found in four sampling points (8, 16, 20, and 21a)—Table 4. However, it should be remembered that TP coefficient in our screening study reflects the total PAHs concentration and fulfils the simple conservative assumption that the measured PAHs are 100% bioavailable (Jensen and Mesman 2006; Umeh et al. 2017). In long-time contaminated soils, hydrocarbons are strongly sorbed and sequestered into organic matter and reveal low bioavailability/bioaccessibility to soil organisms (Maliszewska-Kordybach et al. 2010; Riding et al. 2013; Semple et al. 2013; Klimkowicz-Pawlas et al. 2017), and thus obtained high ChemRI values may lead to the overestimation of risk.

Based on individual LoE risk values, integrated EnvRIs for each sampling point were calculated according to the Triad approach (Eq. 2, Table 5). Chemical LoE risk values were significantly higher than the ecotoxicological and ecological LoE risk factors (Table 4, Table 5) and in 92% affected the integrated EnvRI values, which ranged from 0.44 to 0.94. Only in one sampling point (10a—Table 5) EnvRI (0.44) indicated that there is low risk (0.25 ≤ EnvRI ≤ 0.50)—as it is recommended by Jensen and Mesman (2006) and Dagnino et al. (2008). In few cases (soils 4, 10b and 12), the risk was assessed as low, despite the EnvRIs were slightly above 0.50. These points were located at a significant distance from the main emission sources of PAHs—coking plant and power plant—Fig. 1, and EnvRI was mainly connected with high values of ChemRI factors. It is worth noting that in mentioned soils PAH content was below mean value (616 µg kg^−1^) found in typical Polish agricultural regions (Maliszewska-Kordybach et al. 2008), and potential hazard (HQ value > 1) was revealed by a slight increase in the content, due to only one PAH compound (BbF 102–133 µg kg^−1^; MPC = 100 µg kg^−1^). Additionally, the soil properties such as low pH and C_org_ content affected to a great extent the EnvRI values.

**Table 5:** Chemical, ecotoxicological and ecological risk indexes and the integrated environmental risk (EnvRI)

Risk index	Sampling point														
	4	5	6	8	10a	10b	12	16	17	18	19	20	21	21a	22a
ChemRI	0.86	0.86	0.90	1.00	0.76	0.84	0.89	1.00	0.93	0.98	0.95	1.00	0.99	1.00	0.88
EcotoxRI	0.00	0.00	0.68	0.09	0.27	0.07	0.19	0.02	0.00	0.45	0.10	0.23	0.07	0.37	0.00
EcolRI	0.25	0.40	0.21	0.23	0.00	0.33	0.51	0.00	0.00	0.49	0.00	0.08	0.00	0.07	0.12
EnvRI	0.53	0.56	0.70	0.90	0.44	0.53	0.65	0.94	0.58	0.83	0.64	0.90	0.78	0.94	0.56
SD	0.44	0.43	0.35	0.49	0.38	0.39	0.35	0.57	0.53	0.30	0.52	0.49	0.55	0.42	0.45

*SD* standard deviation

A moderate risk (0.51 ≤ EnvRI ≤ 0.75) was associated with sampling points 5, 6, 17, 19 and 22a located in a distance of 5 to 8 km from the main pollution sources. Almost for all of five soils with moderate risk the level of uncertainty was high; the weight of evidence was strong only for soil 6, as confirmed by the low level of standard deviation—Table 5. On points 17, 19 and 22a, the risk was just indicated by the chemical LoE, as the ecotoxicological and ecological LoE indicated no risk. It was probably related to the high organic matter content (26–73 g kg^−1^) in these soils, which influences the PAHs sorption/desorption processes and limits their bioavailability to soil organisms (Riding et al. 2013; Obrist et al. 2015; Umeh et al. 2017).

For other six soils (8, 16, 18, 20, 21 and 21a), the high values of EnvRI (≥ 0.76) were found, indicating the possibility of adverse environmental effects. As Jensen and Mesman (2006) mentioned, the extremely high risk values force restriction in land use, and only industrial activities in such areas can be carried out. The very high EnvRI above 0.90 calculated for soils 8, 16, 20 and 21a were mainly due to extremely high ChemRI values, while EcotoxRI and EcolRI indicated no or low risk—Table 5. The chemistry LoE produced very high risk estimates, and it was the main reason for relatively large deviation found in the final risk number. It confirms that chemical characterization does not provide specific biological information about potential hazards to organisms (Jensen and Mesman 2006; Dagnino et al. 2008).

## Conclusions

The rural regions are often located in the vicinity of highly urbanized/industrialized areas and may be exposed to emissions of various pollutants, e.g. PAHs. The total concentration of Σ16PAHs ranged from the 376 to 5695 µg kg^−1^ dry weight with median value of 1271 µg kg^−1^. In order to assess the potential ecological risk, the generic approach based on the HQ evaluations was used. PAHs concentration above maximum permissible concentration (HQ 0.01–4.42) was found in sampling points located mainly in the central and north parts of the Czerwionka region, which were exposed to high traffic volume and other various anthropogenic activities such as coke production and mine wastes recovery. To more site-specific evaluations, the first screening stage of Triad procedure including chemical, ecotoxicological and ecological parameters was applied. Different screening bioassays—Rapidtoxkit, Microtox Screening and SPT-Microtox test—were used for the acute toxicity evaluation (Ecotox-LoE). Ecological indexes (Ecol-LoE) comprised microbial parameters related to the soil respiration and dehydrogenases activity.

Based on individual LoE risk values, integrated EnvRIs were calculated according to the Triad approach. EnvRI values ranged from 0.44 to 0.94 and in 92% were affected by ChemRI. Low risk (0.25 ≤ EnvRI ≤ 0.50) was found in soils contaminated only by one PAH (concentration slightly above MPC) and located in a long distance from the main emission sources of PAH (coking plant and power plant). The moderate (EnvRI ≥ 0.51) and high environmental risk (EnvRI ≥ 0.76) observed for 11 sampling points was mainly due to extremely high ChemRI values, while EcotoxRI and EcolRI indicated no or low risk. This high risk may indicate the need to take some remediation action.

Application of the Triad-based evaluations allowed the more realistic assessment and showed a gradient of risk at the sampling sites. However, it should be remembered about high uncertainty associated with the contradictory information given by LoEs. To confirm and avoid overestimation of potential risk, more ecologically relevant bioassays and measurement of the PAH bioavailable fraction should be included in the next tier analysis. Observed evident differences in the risk level may also arise from the weighting method applied in our study. For evaluation of all parameters and individual LoEs, we used equal weighting method, since this method is widely reported for terrestrial environment. Some authors suggest that different weighting factors should be attributed to diverse LoEs risk indexes on the basis of their ecosystem relevance. It seems from our study that in the next Tiers of risk assessment higher weights should be assigned for ecotoxicological and ecological LoEs.
